# Discrimination of alcohol dependence based on the convolutional neural network

**DOI:** 10.1371/journal.pone.0241268

**Published:** 2020-10-27

**Authors:** Fangfang Chen, Meng Xiao, Cheng Chen, Chen Chen, Ziwei Yan, Huijie Han, Shuailei Zhang, Feilong Yue, Rui Gao, Xiaoyi Lv

**Affiliations:** 1 College of Information Science and Engineering, Xinjiang University, Urumqi, Xinjiang, China; 2 The Fourth People's Hospital of Urumqi, Urumqi, Xinjiang, China; 3 School of Pharmacy, Shanghai Jiao Tong University, Shanghai, China; 4 College of Software, Xinjiang University, Urumqi, Xinjiang, China; 5 Key Laboratory of signal detection and processing, Xinjiang University, Urumqi, Xinjiang, China; Northeast Normal University, CHINA

## Abstract

In this paper, a total of 20 sites of single nucleotide polymorphisms (SNPs) on the serotonin 3 receptor A gene (HTR3A) and B gene (HTR3B) are used for feature fusion with age, education and marital status information, and the grid search-support vector machine (GS-SVM), the convolutional neural network (CNN) and the convolutional neural network combined with long and short-term memory (CNN-LSTM) are used to classify and discriminate between alcohol-dependent patients (AD) and the non-alcohol-dependent control group. The results show that 19 SNPs combined with academic qualifications have the best discrimination effect. In the GS-SVM, the area under the receiver operating characteristic (ROC) curve (AUC) is 0.87, the AUC of CNN-LSTM is 0.88, and the performance of the CNN model is the best, with an AUC of 0.92. This study shows that the CNN model can more accurately discriminate AD than the SVM to treat patients in time.

## Introduction

Alcohol dependence is the third most serious public health problem after cardiovascular disease and malignant tumors. Alcohol dependence is a hereditary chronic recurrent disease caused by long-term excessive drinking [1–3]. According to statistics from the National Institute on Alcohol Abuse and Alcoholism (NIAAA), more than 17 million people in the United States abuse/depend on alcohol, causing losses to American society of more than $180 billion [3]. In addition, the World Health Organization estimates that the global mortality rate caused by alcohol from 2000 to 2013 is 4.64% [4], and the global disability rate is 4.15% [1]. A large number of studies have shown that alcohol dependence not only causes damage to personal health, such as cirrhosis [5], gastrointestinal diseases [6,7] and brain aging [8,9], but it also has an impact on an individual’s mental and spiritual well-being in the form of domestic violence [10,11], depression [12], etc. Alcohol dependence severely harms individuals, families, and the social order; therefore, rapid and efficient AD diagnosis methods are indispensable for current clinical diagnosis. However, current clinical diagnostic measures are only based on a combination of clinical interviews, questionnaires, blood tests, etc. [7,13,14]. Although it can reflect the state of patients to some extent, there are problems such as its long cycle and highly uncertain factors [7]; therefore, a more efficient diagnostic tool is needed to support the rapid detection of AD.

SNP refers to a DNA sequence polymorphism caused by the mutation of a single nucleotide in the genome and is one of the most common types of genetic variation in the human genome [15]. Because of its large data volume and uniform distribution, SNPs are widely used in the study of genetics and complex diseases [16], such as its use as a potential biomarker for the diagnosis and treatment of many cancers [15]. In recent years, much research has focused on the SNP information related to diseases [17–19], and certain results have been achieved. Examples include the clinical application of SNP array analysis in early pregnancy abortion [20], and single nucleotide polymorphism loop-mediated isothermal amplification (SNP-LAMP) assisting in the treatment of artemisinin-resistant malaria by decreasing the toxicity in patients due to the time span of medication being too long [21–23]. A study found that the serotonin receptor 5-HT3 plays an important role in the process of rapid neurotransmission in the brain [24]. In addition, the HTR3A and HTR3B genes in the 5-HT3 [25], age [26] and education [27] are closely related to alcohol dependence. In this study, combined with NCBI and literature reports, we selected the SNP information of each of the 10 sites on HTR3A and HTR3B [28–30] combined with the age, education and marital status of the participants as the features of AD diagnosis.

Using machine learning methods for data mining of SNP pathogenic sites has become a research hotspot in the field of bioinformatics [16,31]. Due to the flexibility of modeling different data sources [32], the SVM algorithm is used for many complex diseases [33–35]. In addition, the applicability of deep learning methods to complex diseases has been continuously explored in recent years, especially that of convolutional neural networks [36–39], while CNN-LSTM has achieved good results in medical disease classification [40,41]. However, most studies on alcohol dependence only use genetic information combined with basic mathematical statistics to find genes related to alcohol-dependent patients [25,28]. According to the current survey, this study is the first to propose feature fusion based on SNPs, age, education, and marital status using the GS-SVM and CNN algorithms to conduct an exploratory study on the determination of alcohol-dependent patients. Analogous to other studies that use SNP combined with machine learning to classify diseases [42–44], for example, Yoon et al. used 14 SNPs from 86 cases of coronary heart disease patients and 119 cases of control samples combined with SVM to classify patients with coronary heart disease [45]. Tomida et al. used 6 SNPs from 13 cases of atopic dermatitis patients and 59 cases of control samples combined with artificial neural network (ANN) to distinguish patients with atopic dermatitis [46]. This article collected 154 cases of alcohol-dependent patients and 163 cases of control samples, using the SVM machine learning algorithm combined with grid optimization, the model training is carried out on a combination of 20 SNP sites with age, education and marital status. Among them, after the fusion of 19 SNP sites and the education feature, the overall discrimination effect is the best; and its accuracy, specificity and sensitivity are 87.50%, 91.30% and 83.33%, respectively. After these 20 features are trained by the CNN-LSTM model, the evaluation criteria are more evenly distributed, and their accuracy, specificity and sensitivity are 87.50%, 91.30% and 83.33%, respectively. Through the training of the CNN model using deep learning [47], the classification effect is better, and the accuracy, specificity and sensitivity are 92.05%, 93.48% and 90.48%, respectively.

## 2. Materials and methods

### 2.1 Experimental materials

Participants were recruited to the study from January to May 2017. In this experiment, we collected 154 cases of alcohol-dependent patients who were diagnosed by two chief psychiatrists according to the diagnostic criteria of the Diagnostic and Statistical Manual of Mental Disorders (DSM-IV) [48]. 163 cases of control samples were randomly selected by a psychiatrist using the Alcohol Use Disorders Identification Test (AUDIT) to evaluate the severity of alcohol dependence (> 8 points excluded [49]). All of the 317 samples excluded mental health problems and major disease history. 3–5 ml peripheral blood samples were drawn from each participant, and the collection and demographic data of the whole blood sample were completed by a professional physician at the Fourth People's Hospital of Urumqi.

### 2.2 SNP data

According to the literature report (SNPs associated with alcohol dependence [28,30]), and combined with the NCBI search results, a total of 20 SNPs was selected for our study. They are rs1176722, rs1062613, rs1176713 [30], rs11604247, rs1176719, rs10160548 [29], rs10789970, rs2276305 [29], rs2276302, rs3782025 [29], rs1176746, rs1672717, rs3758987 [28], rs10789980, rs4938056, rs1176744 [29], rs897685, rs1182457, rs145648579 and rs12421126 on HTR3A and HTR3B. The detection of SNP was completed by Shanghai Tianhao Biotechnology Co., Ltd., using the GoldMag® nanoparticle method (Gold Magnet Co., Ltd., Xi’an, China) to extract genomic DNA samples from blood, using Nano drop 2000C (Thermo Scientific, Walther, Massachusetts, USA) to determine DNA concentration, use Sequenom MassARRAY Assay Design 3.0 software (San Diego, California, USA) to design multiple SNP MassEXTEND analysis, use Sequenom MassARRAY RS1000 to genotype SNP (San Diego, California, USA), and use Sequenom Typer 4.0 software (San Diego, California, USA) for data management and analysis. After encoding the detected SNP genotype data and interpolating the missing values [50], in order not to introduce artificial bias, we ensured that the interpolation probability meets the Hardy-Weinberg equilibrium law (HWE) [51].

### 2.3 Data analysis

Before training the model, to make the age, education and marital status evenly distributed for the AD and control groups, statistical analysis was performed to remove outliers. In order not to lose the generality and at the same time obtain a high discriminant accuracy, we choose the Kennard-Stone (KS) algorithm to divide the data, which is widely used in classification problems [52,53] and is based on the Euclidean distance [54]. We use the SVM algorithm (LIBSVM toolbox, MATLAB R2014a) to find the features with the best discriminant effect, and then perform CNN training (Keras, Python 3.7.4) to try to improve the discriminant effect.

The model evaluation indicators of this study are the accuracy, which is the proportion of patients and controls with correct predictions with respect to the total sample; the precision, which is the proportion of patients with correct predictions with respect to the total sample of patients with predictions; the sensitivity, which is the proportion of patients with correct predictions with respect to the total samples of real patients; the specificity, which is the proportion of the control group samples with correct predictions with respect to the real control group samples; and the area under the receiver operating characteristic curve [55]. The relevant code for this study can be obtained from Github (https://github.com/ChenFfha/CNN/).

### 2.4 statement

This study has been approved by the Ethics Committee of the Fourth People's Hospital of Urumqi, Xinjiang. Each participant in the case-control study signed an informed consent form before conducting the experimental test. In addition, this study was conducted in accordance with relevant regulations.

## 3. Results

### 3.1 Data pre-processing and analysis

**Fig 1:**
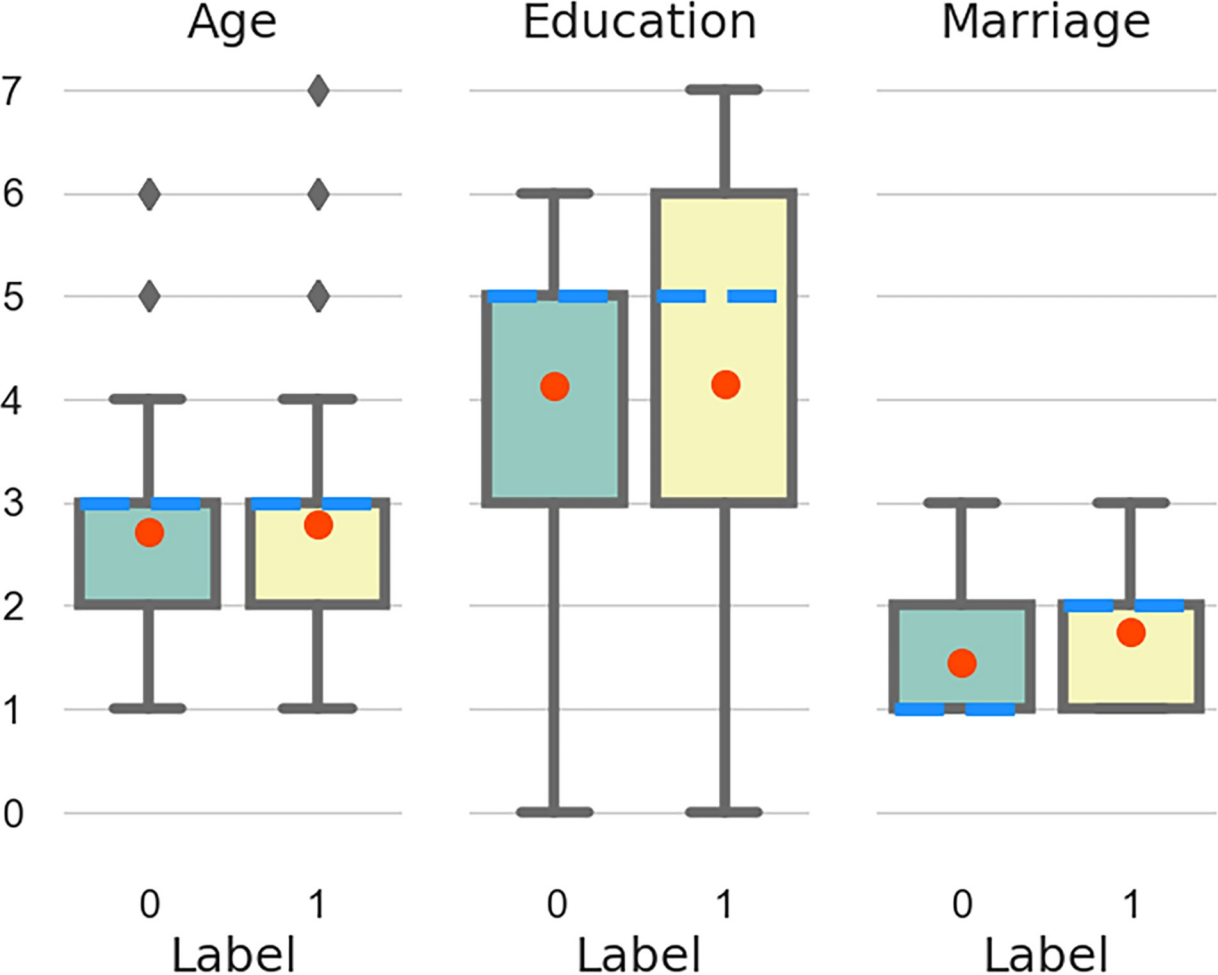
Box-plot. Horizontal axis 0-CONTROL, 1-AD. Education level ranges from 0–7, which represent elementary school to a master’s degree, respectively; marital status ranges from 1–3, which represent married, unmarried and others, respectively; and age ranges from 1–7, which represent that the age is from 24 to 81 years old, quantified at 10 year intervals [57]. The red dot represents the mean, and the blue dotted line represents the median line.

**Fig 2:**
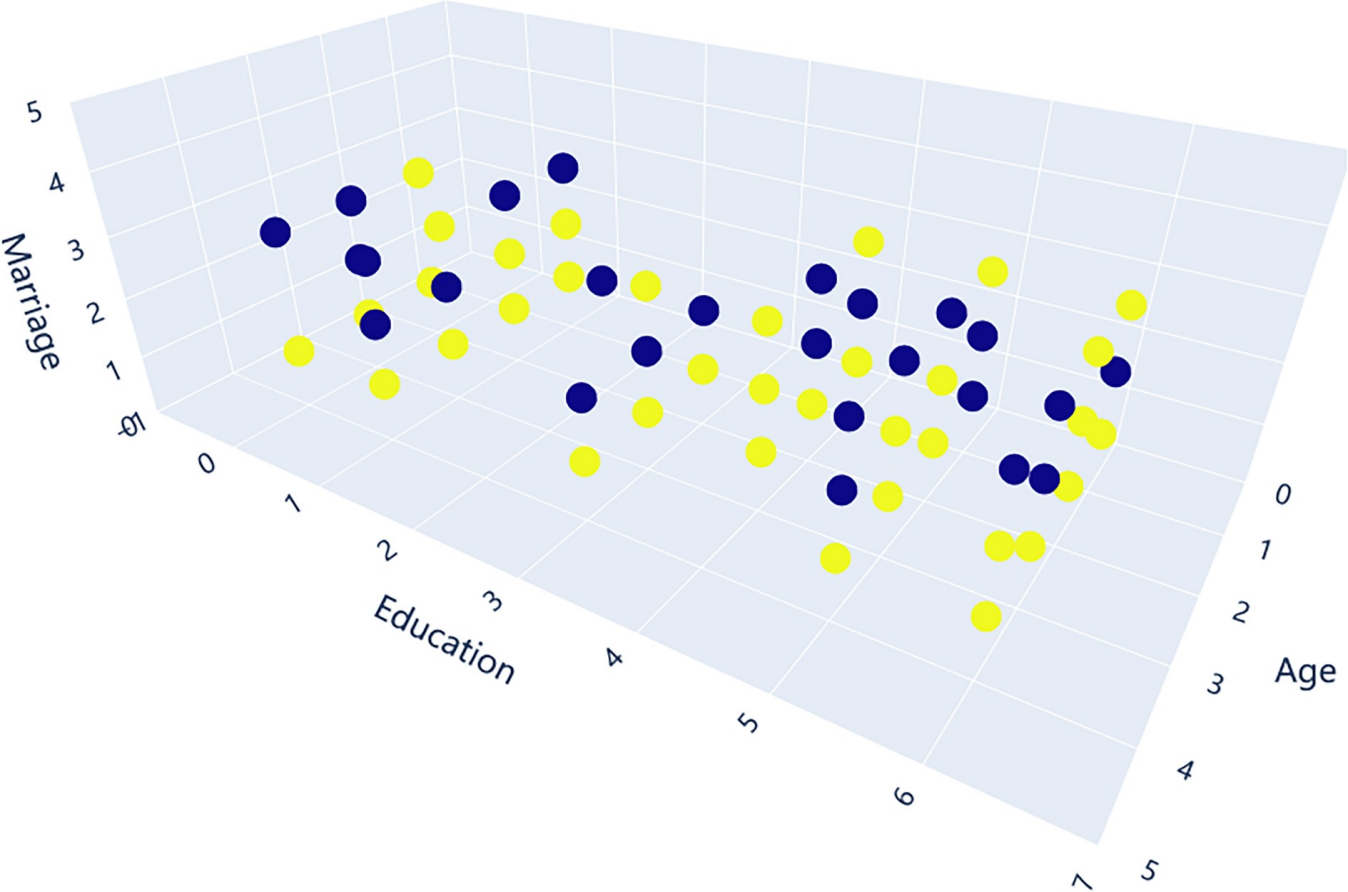
Relationship among age, education and marital status and true label. Blue-AD, Yellow-CONTROL.

To evenly distribute age, education and marital status among the patient and control groups, statistical analysis was performed (Fig 1). Among them, the two highest-educated samples only appeared in the AD group, there were only 18 cases (outliers) between the ages of 60–90 among the recipients, and the distribution was uneven [56]. Remove these data. The details of the participants after excluding outliers are shown in Appendix A. Among the 297 participants, there are 141 cases of alcohol-dependent patients (AD001-AD141) and 156 cases of control samples (CONTROL001- CONTROL 156). Their age is concentrated in the 24–59 years old, in order to facilitate observation, we set the age of 21–30 years old as 1, and so on, set the age of 50–60 years old as 4. In addition, the participants were educated from primary school to undergraduate, taking into account married, unmarried and other marital situations. Visualizing these samples (Fig 2), we can see that age, education and marital status are almost evenly distributed in AD and control groups. In alcohol dependent patients and control groups, we considered people of all ages, as well as their education and marital status, so our data are representative.

**Fig 3:**
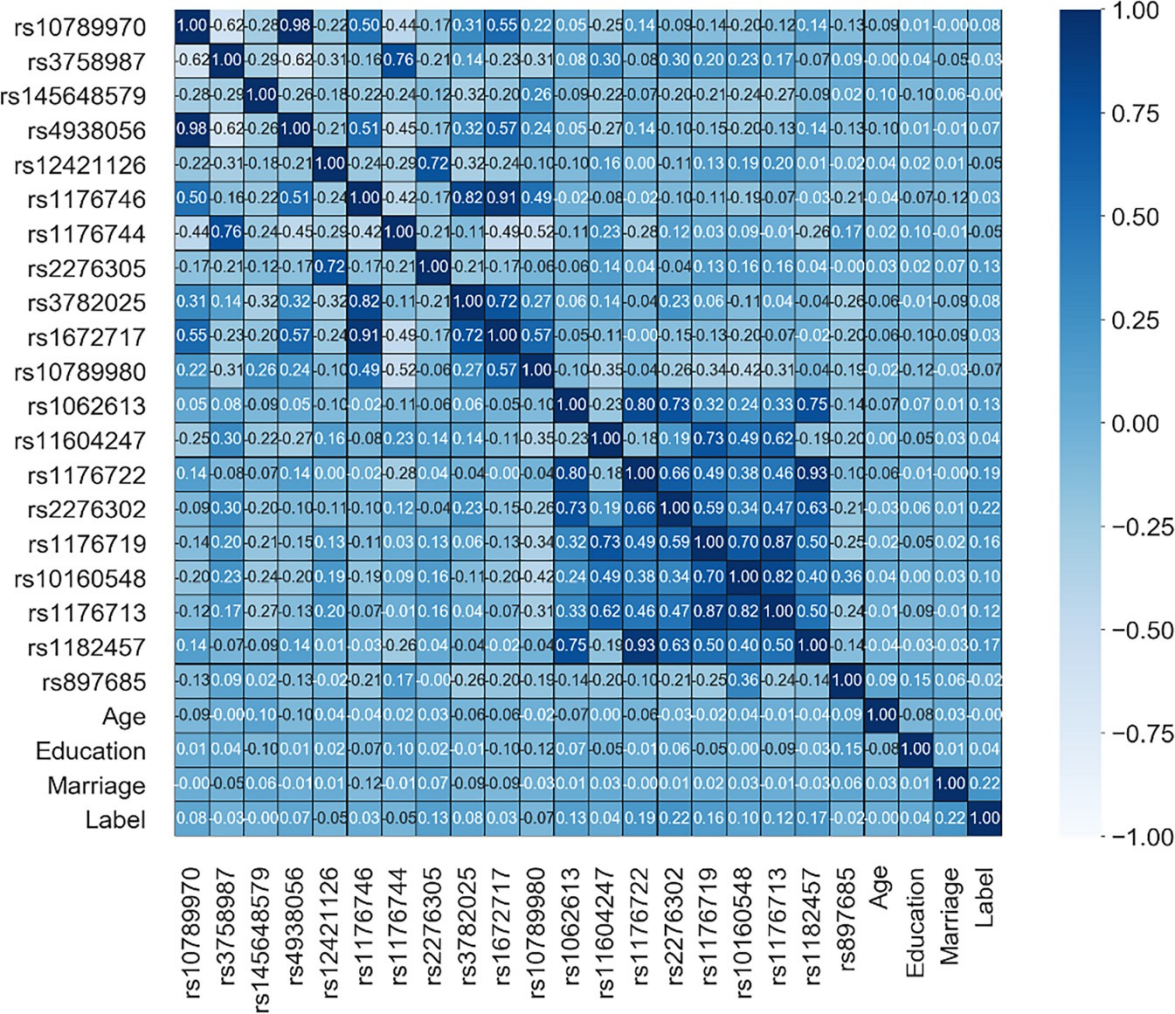
Pearson correlation coefficients. Strong correlations are rs4938056 and rs10789970 (0.98), rs1176746 and rs1672717 (0.91) and rs3782025 (0.82), rs1176722 and rs1182457 (0.93) and rs1062613 (0.80), and rs1176713 and rs1176719 (0.87) and rs10160548 (0.82).

**Fig 4:**
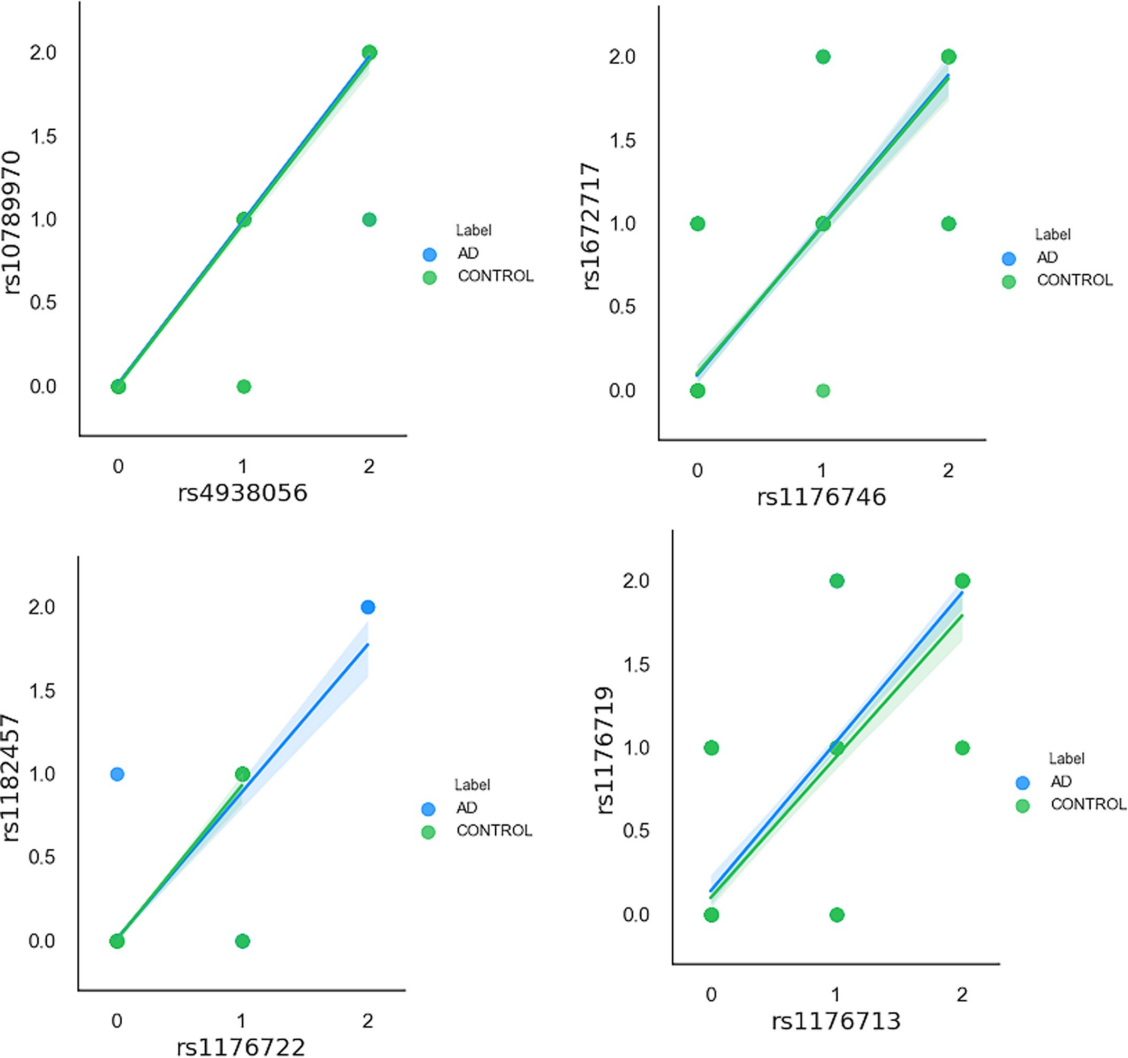
Linear regression plots. Correlation coefficient> 0.85.

The SNP data of these 297 samples, including the 141 AD and 156 control groups, were encoded using the PLINK command as genotype AA coded 0, genotype AB coded 1, and genotype BB coded 2 [58], and then the missing values of the encoded data set were interpolated at random. Fig 3 calculates the Pearson correlation coefficients for the data after removing the outliers. It can be seen from the figure that the correlation among most features is very low, while rs4938056 and rs10789970 are the highest at 0.98. In the linear regression plots of the SNP sites (Fig 4), the correlation coefficient is greater than 0.8 [59], that is, it has a very strong correlation, and only rs4938056 and rs10789970 are correlated. The correlation coefficient is 0.98, and it has a certain linear correlation [60].

Before the model training, the KS algorithm was used to extract 70% of the data from the control group and AD group as the training set, including 110 cases from the control group and 99 cases of from the AD group. The remaining 42 cases of the control group and 46 cases of the AD data are the test set.

### 3.2 SVM model

**Fig 5:**
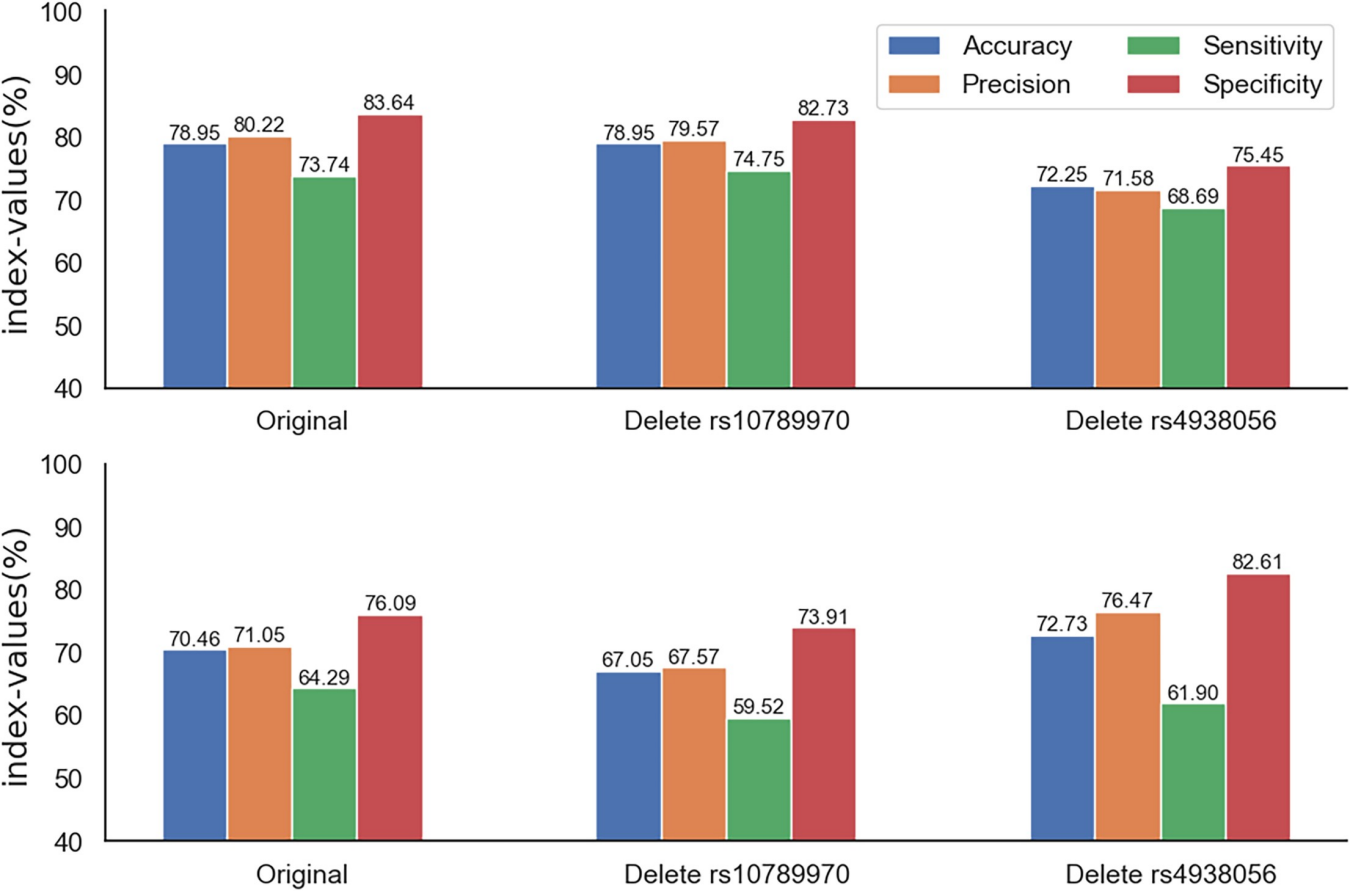
SVM training results of the different genotype data. The top picture is the result of the training set, and the bottom picture is the result of the test set.

From 3.1, we know that rs4938056 and rs10789970 have a very strong correlation. For the 20 SNPs, the 19 SNPs with rs4938056 removed and the 19 SNPs with rs10789970 removed, we divide the data set. We set the initial penalty parameter C as [2^−10^,2^10^], the range of kernel function parameter g is set as [2^−10^,2^10^], and the grid search parameters are optimized through five-fold cross-validation. Finally, the SVM model is trained, and the results are shown in Fig 5. The original genotype data results are overfit, and the accuracy is only 70.46%. When we remove rs10789970, the results do not change much. When excluding rs4938056, the overfitting phenomenon is suppressed, the accuracy is 72.73%, the specificity is 82.61%, the sensitivity is 61.90%, and the overall effect is better. At this time, the best C is 181.019, the best g is 0.004, and the kernel function is the Gaussian kernel function.

The SNPs after excluding rs4938056 is combined with age, education and marital status and input into the GS-SVM model. Various model evaluation indicators (Table 1) show that the discrimination accuracy has been improved by the feature fusion method, but the sensitivity of the SNPs combined with age is low. Although the accuracy of combined education and marital status is the highest at 88.64%, its sensitivity is only 80.95%, and the distribution of the various indicators is uneven (± 14.7). In contrast, the overall discrimination effect of SNPs combined with academic qualifications is the best, with an accuracy, a specificity and a sensitivity of 87.50%, 91.30% and 83.33%, respectively; and the distribution of various indicators is relatively uniform (± 7.97).

**Table 1:** Evaluation index.

Features	Accuracy (%)	Precision (%)	Sensitivity (%)	Specificity (%)
SNP	72.73	76.47	61.90	82.61
SNP&A	72.73	80.00	57.14	86.96
SNP&E	87.50	89.74	83.33	91.30
SNP&M	76.14	81.82	64.29	86.96
SNP&A E	81.82	96.43	64.29	97.83
SNP&A M	71.59	79.31	54.76	86.96
SNP&E M	88.64	94.44	80.95	95.65
SNP&A E M	79.55	83.33	71.43	86.96

Note: A = Age, E = Education, and M = Marital status. SNP is the genotype data after excluding rs4938056.

### 3.3 CNN model

It can be seen from 3.2 that the sensitivity of the SNPs and academic qualifications needs to be improved after training using the GS-SVM model. To obtain a better discrimination effect, we used the convolutional layer and the pooling layer to try to extract more effective features by adjusting the GoogLeNet model [61], thereby improving the patient discrimination accuracy.

**Fig 6:**
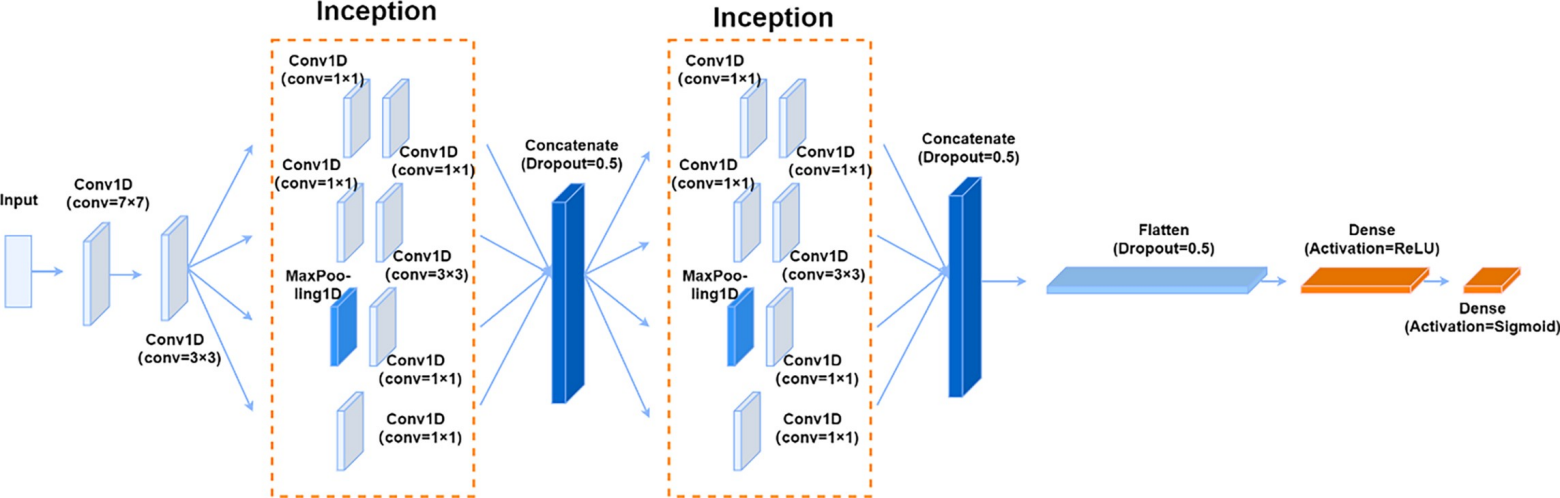
CNN model. (Add batch normalization after each convolutional layer). a. Indicators of various model items. b. ROC curve.

The 19 SNPs and educational background features after excluding rs4938056 from the original data are used as the input of the GoodLeNet model. The adjusted model structure is like that shown in Fig 6. To prevent the disappearing/exploding gradient and overfitting, batch normalization is added after each convolutional layer [62], and a dropout layer with a discarding rate of 0.5 is added to the fully connected layer [63]. The number of epochs in the model is 200, the batch size is 32, the learning rate is 0.01, the loss function is the cross-entropy loss suitable for binary classification, and the optimizer that is chosen is Adam, which performs well in practice [64]. The results showed that its accuracy, precision, specificity and sensitivity were 92.05%, 92.68%, 90.48% and 93.48%, respectively.

### 3.4 CNN-LSTM model

In recent years, the CNN-LSTM model has performed well in classification tasks [65–67]. This article adds LSTM after the second Inception block in Fig 6 is fully connected, sets the number of hidden layer units to 512, adds L2 regularization to prevent overfitting, and sets the regularization parameter to 0.005. The results showed that its accuracy, precision, specificity and sensitivity were 87.50%, 86.05%, 88.10% and 86.96%, respectively.

### 3.5 Comparison of three models

**Fig 7:**
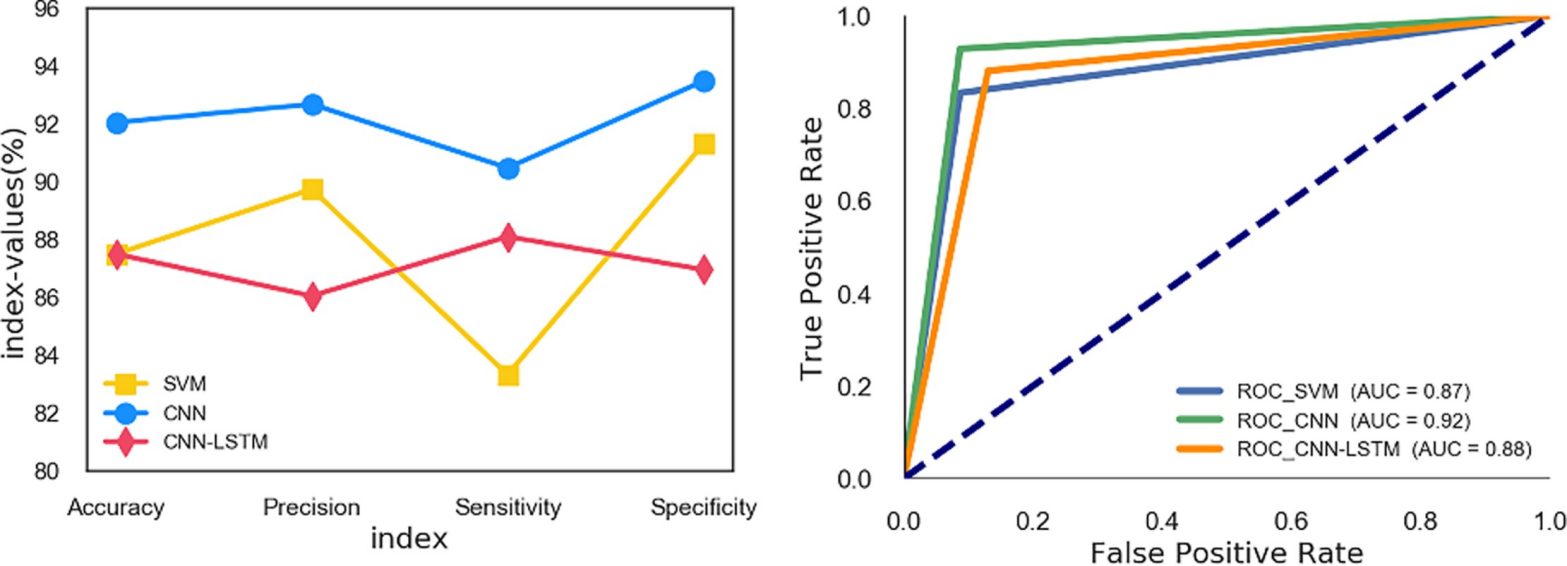
GS-SVM and CNN model index comparison.

From the comparison of various model indicators of the three models in Fig 7, it can be seen that the various indicators of SVM are unevenly distributed, with the highest specificity and the lowest sensitivity, 91.30% and 83.33% respectively. Compared with SVM, the four model evaluation indicators in CNN-LSTM fluctuate less, and its accuracy is the same as SVM, which is 87.50%. The CNN model makes the accuracy, specificity and sensitivity reach 92.05%, 93.48% and 90.48% respectively. Compared with GS-SVM and CNN-LSTM, the AUC value of the CNN model reaches 0.92, which means that it has a better overall discrimination effect.

## 4. Discussion

Here, we propose a method that uses the SNP on HTR3A and HTR3B, age, education level and marital status of human combined with convolutional neural network to screen for patients with alcohol dependence, which is of great significance for the prevention and treatment of related diseases caused by alcohol dependence. However, the participants in this study were all male and didn’t analyze women. In addition, participants' educational background was all in primary school, junior high school, technical school, technical secondary school, senior high school, junior college and undergraduate. and other educational background groups were not studied.

## 5. Conclusions and prospects

In this study, we used SNPs of HTR3A and HTR3B to fuse the features with age, education level and marital status, and we used the GS-SVM, CNN and CNN-LSTM models to discriminate patients. Among them, rs10789970 has a strong correlation with rs4938056, and the combination of SNPs excluding rs10789970 and academic qualifications can effectively distinguish AD, and the accuracy, specificity and sensitivity are 87.50%, 91.30% and 83.33%, respectively. By adjusting the GoogLeNet model for training, the various performance indicators have been improved. The accuracy, specificity and sensitivity are 92.05%, 93.48% and 90.48%, respectively, and the distribution is relatively uniform. The classification effect of CNN-LSTM model is slightly better than SVM, but it is worse than CNN. This study is a preliminary exploratory study of alcohol-dependent patients. There are still some disadvantages, such as the small sample size. In follow-up research, we will add phenotypic traits (blood type, weight, etc.) for feature fusion, and establish a CNN rapid discrimination AD model based on SNPs, education, and phenotypic trait information.

## Appendix A. Summary table

**Table:** 

Number	Age	Education level	Marital status
AD001	4	0	1
AD002	2	1	1
AD003	1	6	2
AD004	2	4	2
AD005	2	0	3
AD006	3	3	1
AD007	4	5	3
AD008	4	1	2
AD009	3	4	1
AD010	2	6	2
AD011	1	1	2
AD012	1	4	1
AD013	1	1	2
AD014	2	5	2
AD015	4	1	2
AD016	2	4	1
AD017	1	5	2
AD018	3	5	2
AD019	2	1	3
AD020	3	6	1
AD021	2	5	3
AD022	3	4	1
AD023	2	1	1
AD024	4	3	1
AD025	3	0	2
AD026	3	5	1
AD027	4	6	3
AD028	4	6	1
AD029	3	3	3
AD030	3	1	2
AD031	1	4	2
AD032	2	5	1
AD033	3	6	1
AD034	3	6	3
AD035	3	5	2
AD036	2	6	2
AD037	3	5	3
AD038	4	3	2
AD039	1	4	2
AD040	4	1	1
AD041	2	6	3
AD042	3	5	1
AD043	3	1	2
AD044	3	5	2
AD045	3	5	2
AD046	2	4	3
AD047	3	1	1
AD048	2	6	1
AD049	1	5	2
AD050	2	5	1
AD051	1	1	3
AD052	3	5	3
AD053	3	6	1
AD054	2	4	2
AD055	2	2	2
AD056	3	3	1
AD057	3	6	2
AD058	3	5	3
AD059	2	4	2
AD060	2	6	1
AD061	2	6	1
AD062	1	6	1
AD063	4	6	3
AD064	2	1	2
AD065	3	4	1
AD066	3	5	2
AD067	3	6	1
AD068	2	6	1
AD069	3	4	2
AD070	2	4	1
AD071	3	1	2
AD072	3	3	1
AD073	4	6	3
AD074	4	0	3
AD075	3	6	1
AD076	2	6	3
AD077	2	5	2
AD078	2	4	2
AD079	2	6	1
AD080	3	5	2
AD081	2	6	2
AD082	3	4	1
AD083	2	5	1
AD084	3	4	2
AD085	1	1	1
AD086	2	6	1
AD087	2	5	1
AD088	3	5	1
AD089	1	5	1
AD090	3	0	3
AD091	3	3	2
AD092	4	1	2
AD093	4	1	3
AD094	1	5	1
AD095	4	0	1
AD096	3	5	3
AD097	3	1	1
AD098	2	6	2
AD099	4	5	2
AD100	3	5	2
AD101	3	5	2
AD102	3	5	2
AD103	1	5	2
AD104	4	6	3
AD105	3	5	1
AD106	3	5	2
AD107	3	5	2
AD108	2	5	1
AD109	2	5	1
AD110	3	5	1
AD111	3	5	2
AD112	2	6	1
AD113	2	5	1
AD114	2	3	2
AD115	4	5	2
AD116	3	6	1
AD117	2	6	1
AD118	2	5	2
AD119	3	5	1
AD120	2	6	2
AD121	2	6	1
AD122	3	6	3
AD123	3	5	2
AD124	2	5	3
AD125	3	5	2
AD126	2	5	2
AD127	3	3	2
AD128	2	5	1
AD129	2	5	2
AD130	3	6	1
AD131	3	5	2
AD132	3	6	1
AD133	2	5	2
AD134	3	1	2
AD135	3	5	1
AD136	2	6	1
AD137	1	5	1
AD138	2	4	3
AD139	2	6	2
AD140	3	6	2
AD141	4	1	2
CONTROL001	1	1	1
CONTROL002	2	6	1
CONTROL003	4	0	1
CONTROL004	2	5	3
CONTROL005	3	3	1
CONTROL006	2	0	3
CONTROL007	3	5	3
CONTROL008	2	5	1
CONTROL009	3	5	2
CONTROL010	4	1	1
CONTROL011	1	1	2
CONTROL012	1	4	1
CONTROL013	2	5	1
CONTROL014	1	4	3
CONTROL015	3	6	2
CONTROL016	3	5	1
CONTROL017	4	1	1
CONTROL018	3	6	2
CONTROL019	3	3	1
CONTROL020	4	0	1
CONTROL021	2	1	2
CONTROL022	3	3	3
CONTROL023	1	4	1
CONTROL024	4	6	3
CONTROL025	1	6	3
CONTROL026	2	1	1
CONTROL027	3	1	3
CONTROL028	3	3	1
CONTROL029	2	0	1
CONTROL030	3	0	1
CONTROL031	3	5	2
CONTROL032	2	6	1
CONTROL033	3	4	2
CONTROL034	4	6	2
CONTROL035	3	5	1
CONTROL036	3	1	1
CONTROL037	2	6	1
CONTROL038	3	3	3
CONTROL039	3	1	2
CONTROL040	3	5	1
CONTROL041	4	6	1
CONTROL042	3	4	1
CONTROL043	3	5	3
CONTROL044	2	6	3
CONTROL045	3	3	2
CONTROL046	3	3	1
CONTROL047	4	6	2
CONTROL048	2	6	2
CONTROL049	2	5	1
CONTROL050	1	5	3
CONTROL051	2	4	3
CONTROL052	3	4	3
CONTROL053	4	5	2
CONTROL054	3	4	2
CONTROL055	2	3	1
CONTROL056	1	1	1
CONTROL057	2	4	3
CONTROL058	2	5	1
CONTROL059	1	5	1
CONTROL060	3	6	1
CONTROL061	3	5	2
CONTROL062	2	5	1
CONTROL063	4	6	1
CONTROL064	3	3	1
CONTROL065	1	5	2
CONTROL066	1	6	1
CONTROL067	2	5	1
CONTROL068	3	5	1
CONTROL069	3	5	1
CONTROL070	2	5	2
CONTROL071	4	3	1
CONTROL072	3	5	1
CONTROL073	2	3	1
CONTROL074	3	3	1
CONTROL075	3	5	1
CONTROL076	2	5	1
CONTROL077	2	5	3
CONTROL078	1	4	1
CONTROL079	2	1	1
CONTROL080	2	4	1
CONTROL081	3	3	2
CONTROL082	3	5	1
CONTROL083	3	5	1
CONTROL084	1	1	1
CONTROL085	2	6	1
CONTROL086	1	1	2
CONTROL087	3	6	2
CONTROL088	4	5	2
CONTROL089	3	1	1
CONTROL090	2	5	1
CONTROL091	2	4	1
CONTROL092	3	1	1
CONTROL093	3	1	1
CONTROL094	2	6	2
CONTROL095	2	4	1
CONTROL096	3	5	1
CONTROL097	2	5	3
CONTROL098	2	5	1
CONTROL099	4	1	1
CONTROL100	4	6	1
CONTROL101	1	5	1
CONTROL102	3	4	1
CONTROL103	3	1	1
CONTROL104	3	4	2
CONTROL105	3	5	1
CONTROL106	2	5	1
CONTROL107	3	4	1
CONTROL108	2	5	1
CONTROL109	3	5	1
CONTROL110	4	6	1
CONTROL111	3	4	2
CONTROL112	3	3	1
CONTROL113	3	1	1
CONTROL114	2	5	1
CONTROL115	2	0	1
CONTROL116	3	5	1
CONTROL117	3	5	1
CONTROL118	2	6	1
CONTROL119	2	5	2
CONTROL120	4	6	1
CONTROL121	2	6	2
CONTROL122	2	5	2
CONTROL123	3	5	1
CONTROL124	3	4	3
CONTROL125	2	5	2
CONTROL126	3	5	1
CONTROL127	3	5	2
CONTROL128	2	5	2
CONTROL129	3	5	2
CONTROL130	4	5	1
CONTROL131	3	5	1
CONTROL132	3	6	1
CONTROL133	2	5	1
CONTROL134	3	4	1
CONTROL135	3	5	1
CONTROL136	2	6	1
CONTROL137	2	4	1
CONTROL138	1	1	1
CONTROL139	3	3	1
CONTROL140	3	3	1
CONTROL141	3	6	1
CONTROL142	2	3	1
CONTROL143	3	1	1
CONTROL144	3	4	1
CONTROL145	3	4	1
CONTROL146	3	5	2
CONTROL147	3	5	1
CONTROL148	3	4	1
CONTROL149	3	5	1
CONTROL150	3	4	1
CONTROL151	2	6	1
CONTROL152	2	6	2
CONTROL153	3	3	1
CONTROL154	2	6	1
CONTROL155	2	5	1
CONTROL156	2	6	1

## Supporting information

S1 Dataset (ZIP)
